# Decreased Medial Prefrontal Cortex Glutamate Levels in Perimenopausal Women

**DOI:** 10.3389/fpsyt.2021.763562

**Published:** 2021-12-13

**Authors:** Sidney Yap, Jessica Luki, Christopher C. Hanstock, Peter Seres, Tami Shandro, Sarah E. C. Hanstock, Alynna Lirette, Huaying (Helen) Zhao, Katherine J. Aitchison, Jean-Michel Le Melledo

**Affiliations:** ^1^Department of Psychiatry, University of Alberta, Edmonton, AB, Canada; ^2^Department of Biomedical Engineering, University of Alberta, Edmonton, AB, Canada; ^3^Lois Hole Hospital for Women, Royal Alexandra Hospital, Edmonton, AB, Canada; ^4^Department of Medical Genetics, University of Alberta, Edmonton, AB, Canada; ^5^The Neuroscience and Mental Health Institute, University of Alberta, Edmonton, AB, Canada; ^6^Edmonton Mood and Anxiety Disorders Program, University of Alberta Hospital, Edmonton, AB, Canada

**Keywords:** perimenopause, glutamate, medial prefrontal cortex, depression, magnetic resonance spectroscopy - MRS

## Abstract

**Objective:** There is an increased risk of experiencing depression during perimenopause (PM), a period of rapidly changing female hormone concentrations. Women at particular risk of developing major depression (MD) during PM are those with history of mood sensitivity to female hormone fluctuations i.e., women with a history of premenstrual dysphoric disorder (PMDD) and/or post-partum depression (PPD). Depressive symptomology has been associated with fluctuations of glutamate (Glu) levels in the medial prefrontal cortex (MPFC) in MD patients as well as PMDD and PPD patients. The objective of the study was to compare MPFC Glu levels in healthy perimenopausal and reproductive-aged (RD) women.

**Methods:** Medial prefrontal cortex Glu levels in healthy perimenopausal (*n* = 15) and healthy RD women (*n* = 16) were compared *via* Magnetic Resonance Spectroscopy (MRS) scan using a 3 Tesla (T) magnet. Absence of depressive symptomology and psychiatric comorbidity was confirmed *via* semi-structured interview. Participants were scanned during the early follicular phase (FP) of the menstrual cycle (MC).

**Results:** Mean MPFC Glu concentrations were decreased in the PM group compared to RD group (PM mean = 0.57 ± 0.03, RD mean = 0.63 ± 0.06, *t* = −3.84, *df* = 23.97, *p* = 0.001).

**Conclusion:** Perimenopause is associated with decreases in MPFC Glu levels. This decrease may be contributing to the increased risk of experiencing depression during PM. Further research should assess MPFC Glu levels in perimenopausal women suffering from MD.

## Introduction

The perimenopause (PM) is a phase within the normal female reproductive life cycle marked by the end of menstrual cycle (MC) regularity. The average age of PM onset is 46 years old and typically lasts 5 years (1).

Risk factors for PM depression include a history of major depression (MD) or a history of mood sensitivity to female hormone fluctuations [i.e., a history of premenstrual dysphoric disorder (PMDD) or post-partum depression (PPD)] (2, 3).

Although the peripheral physiological alterations associated with the PM are well-understood, little is known regarding the impact of PM on brain biology. Without such knowledge, it is difficult to investigate the cerebral mechanisms responsible for the increased risk of MD during PM.

Glutamate (Glu), the major excitatory neurotransmitter, is widespread throughout the brain functioning at approximately 60% of the synapses within the central nervous system (4). An increasing body of evidence supports the involvement of the glutamatergic system in the pathophysiology of MD (5).

The medial prefrontal cortex (MPFC) has been found to be one of the critical brain regions involved in the pathogenesis of MD, playing a crucial role in the affective and cognitive deficits of MD (6).

Magnetic resonance spectroscopy (MRS) is the only non-invasive neuroimaging technique which allows for the direct detection and measurement of metabolite concentration levels, such as Glu, in localized brain regions (7). Additionally, magnetic resonance imaging (MRI) performed alongside MRS is used to measure and control for brain tissue composition, allowing for the proportions of gray matter (GM), white matter (WM), and cerebrospinal fluid (CSF) to be determined and to assess whether changes in tissue composition affect the measurements of neurometabolites.

Previous MRS investigations have found decreased MPFC Glu concentrations in MD patients (8). Our research group previously found a decrease in MPFC Glu levels from the follicular phase (FP) to the luteal phase (LP) of the MC (9), suggesting that fluctuations in female hormones impact MPFC Glu levels. We also found increased MPFC Glu levels in women suffering from PPD (10), suggesting that MPFC Glu levels may play a role in episodes of MD occurring in the context of female hormone fluctuations.

It was therefore logical for our research group to investigate whether PM, which, like post-partum, is a period linked to increased risk of experiencing an episode of MD and is associated with unique and unpredictable rapid changes in female hormone levels, was associated with alterations in MPFC Glu levels.

The objective of the MRS study presented here was to compare MPFC Glu levels in healthy PM and healthy reproductive-aged (RD) women. Our hypothesis was that MPFC Glu levels referenced to Creatine (Cr) would be decreased in PM women and that this decrease may be a risk factor for the development of future MD.

## Methods

### Participants

Perimenopausal (*n* = 15) and RD (*n* = 16) physically and mentally healthy women 18 years of age and older were recruited for the study. The study protocol was approved by the Health Research Ethics Board of the University of Alberta and conducted in accordance with the Declaration of Helsinki. Written informed consent was collected from all participants. Following collection of informed consent, participants took part in two visits: a screening visit and a scanning visit.

#### Inclusion Criteria, Both Age Groups

Physically and mentally healthy women 18 years of age and older and use of a satisfactory birth control method that does not deliver female hormones.

#### Inclusion Criteria, RD Group

Regular occurrence of MC.

#### Inclusion Criteria, PM Group

Undergoing PM, with menopausal status being defined as either early perimenopausal, if menstrual period had occurred in the past 3 months with changes in regularity over the previous 12 months, or late perimenopausal, if no menstrual period was experienced within the past 3 months but some menstrual bleeding was experienced within the past 12 months. The classifications are like those recommended by the World Health Organization and the Stages of Reproductive Aging Workshop (11, 12).

#### Exclusion Criteria for all Subjects

(1) Current or lifetime history of any psychiatric illness [confirmed using the Mini-International Neuropsychiatric Interview (MINI) based on Diagnostic and Statistical Manual of Mental Disorders-5 criteria] (13); (2) any contraindications to MRI; (3) any medical condition that would interfere with the study e.g., endocrine or neurological condition (14); or pregnancy; (4) intake of psychotropic drugs or other medication that may impact brain metabolite levels or brain water content at any time while participating in the study (the minimum length of abstinence for any psychotropic drug or other medication that may impact brain metabolite levels or brain water content was 3 months); (5) attending formal psychotherapy sessions or the use of self-help books; (6) high suicidal risk; (7) use of illicit or recreational drugs; (8) dependence on alcohol and tobacco products or excessive coffee consumption (15); and (9) use of birth control methods which deliver female hormones.

### Study Protocol

Following phone interview, individuals who appeared to be eligible were scheduled for a screening visit. This visit consisted of taking a complete medical and psychiatric history of the participant. Lack of psychiatric illness was verified using the MINI (13). Women who met inclusion and exclusion criteria were scheduled for a scanning visit. The scanning visit was completed between day 2 and 6 of the FP of the MC. All participants underwent an MRS scan and completed Beck's Depression Inventory (BDI), while PM participants were also administered the Greene climacteric scale (GCS) and menopause rating scale (MeRS) to assess PM-related symptomology. In addition, a blood sample used to measure plasma estradiol and progesterone levels was collected from all participants. These samples were collected at the outpatient laboratory located in the Kaye Edmonton Clinic. Hormone measurements were conducted at the Alberta Precision Laboratories at the University of Alberta Hospital. Third generation Elecsys^®^ immunoassay (Roche Diagnostics) was used to measure plasma estradiol, while Access Progesterone assay (Beckman Coulter) was used to measure plasma progesterone.

### Magnetic Resonance Spectroscopy and Imaging

**Figure 1:**
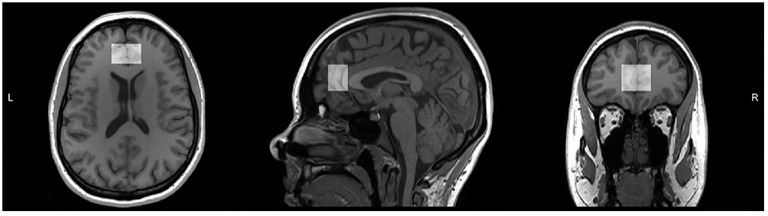
T1 weighted magnetic resonance image of one study participant from reproductive-aged group with medial prefrontal cortex voxel highlighted in transverse, sagittal, and coronal views. Voxel position and orientation were prescribed so that voxel is perpendicular to and centered on midline in transverse and coronal views and parallel to and aligned with corpus callosum line in sagittal view.

Magnetic resonance data was collected at the Peter S. Allen MR Research Center, University of Alberta, Edmonton, Canada, using a Siemens Prisma 3 Tesla (T) scanner (Erlangen, Germany) equipped with a 64-channel head-neck coil for signal reception. Anatomical images were acquired using sagittal 3D T1-weighted magnetization-prepared rapid gradient-echo (MPRAGE) sequence in 3 min 39 s (TR: 1,800 ms, TE: 2.37 ms, TI: 900 ms, flip angle: 8, field of view: 250 × 250 mm, image matrix: 288 × 288, slice thickness: 0.85 mm, number of slices: 208, resolution: 0.87 × 0.87 × 0.85 mm, parallel acceleration factor: 3). Voxel position and orientation were prescribed so that the voxel is perpendicular to and centered on midline in transverse and coronal views and parallel to and aligned with corpus callosum line in sagittal view (Figure 1).

**Figure 2:**
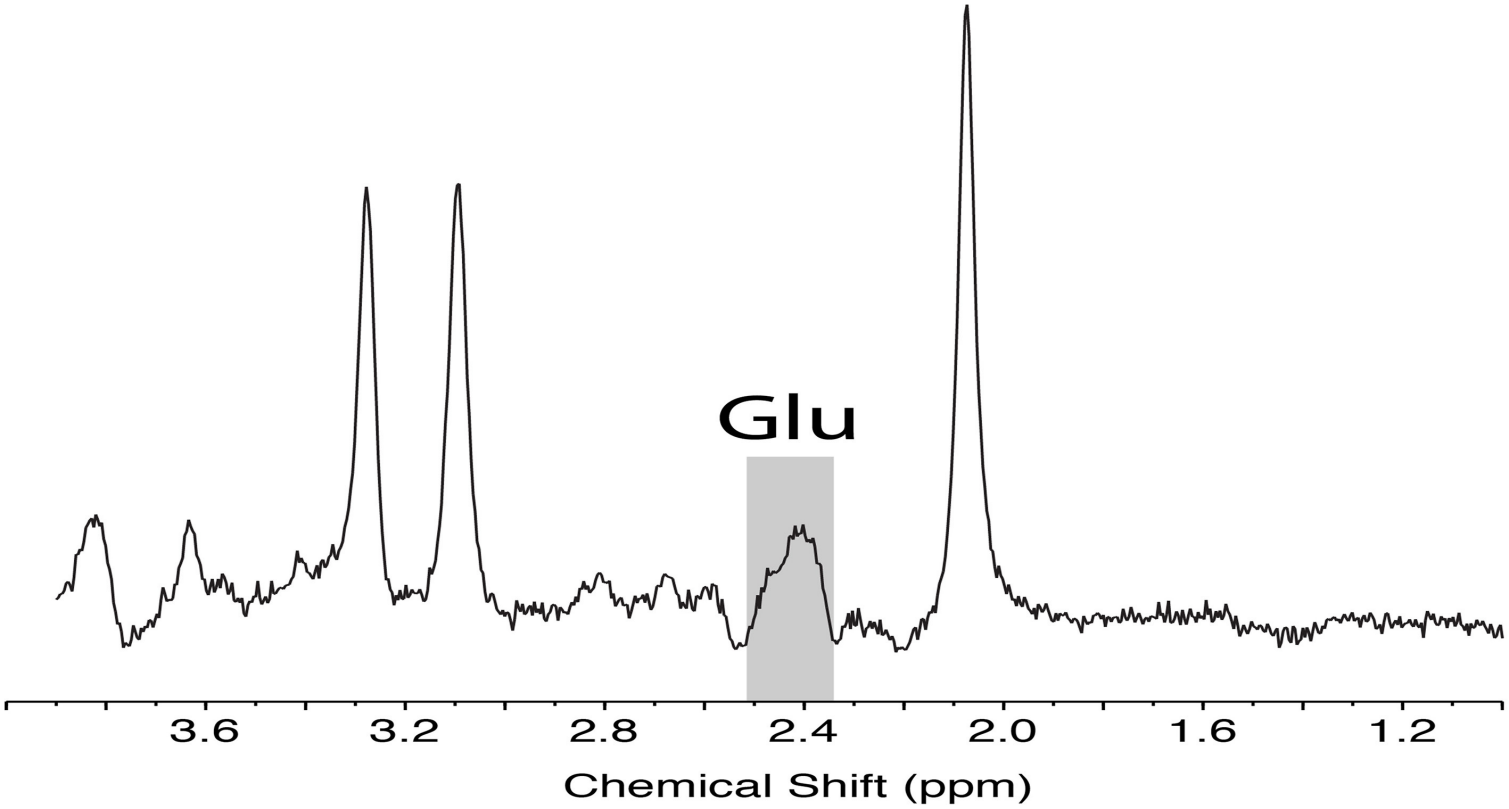
Representative PRESS localized MRS data acquired from the medial prefrontal cortex with sequence timings optimized for recovering signal from glutamate (TE1, TE2 = 90, 18 ms). Spectra illustrates the unfiltered data with Glu peak highlighted. Glu, glutamate; MRS, magnetic resonance spectroscopy, PRESS, point resolved spectroscopy.

These images were used for planning the position of spectroscopy voxels as well as for volumetric and segmentation analysis. Point resolved spectroscopy (PRESS) was used to isolate measurements of Glu from Glutamix (Glx), a mix of Glu and glutamine, following the methods outlined by Harris et al. (16). A sample PRESS spectrum is shown in Figure 2.

Adequate signal-to-noise ratio (SNR) was obtained by acquiring data from the voxel positioned in the MPFC (25 × 20 × 30 mm^3^) summing 64 averages, repeated twice, in 5 min 45 s (PRESS:TR: 2,500 ms, TE1, 2:90, 18 ms). Automated metabolite quantification of the proton MR spectra was performed using LCModel^®^ (version 6.3-1L) (17). Manual inspection was conducted on each spectrum fitted by LCModel to assure quality with respect to line-shape, line-width, SNR, and the presence of spurious signal as a result of eddy current effects. Spectra not meeting these quality check criteria or those with an LCModel fit with a standard deviation >15% were excluded from further analysis. Statistical Parametric Mapping (SPM12) (18) and Gannet software (version 3.0, http://www.gabamrs.com) were used for T1 image volumetric and segmentation analysis. Components of Gannet package were used to generate a mask of the MRS voxel in T1-image space and to utilize the SPM12 “Segmentation” function to derive GM, WM, and CSF voxel fractions.

Magnetic resonance spectroscopy quality is particularly sensitive to participant motion; the acquisition of two spectra greatly reduced the possibility of attaining unusable metabolite data because of brief movements during the scanning period. The peak area estimates from LCModel of Glu were of interest for this study. The final ratio of MPFC Glu to Cr was the average of the ratios derived from the two scans in that region. Acquisition of two spectra allowed for averaging of small fluctuations in metabolite peak measures, thereby arriving at more accurate results for each participant.

Magnetic resonance spectroscopy data for two participants were affected by motion; these participants were scanned again between day 2 and 6 of FP, consistent with study protocol. In two participants one of the two acquired spectra were excluded from further analysis based on manual inspection of spectra quality. These participants were not scanned again, however, as one of the spectra acquired from each participant was of adequate quality.

### Statistical Analysis

Statistical analysis was performed using the IBM Statistical Package for Social Sciences software for Windows Version 26.0 (SPSS 26.0) (IBM Corp., Armonk, NY, USA). The data in this study was normally distributed. A two-tailed *t*-test was used for independent sample analysis of variables between PM and RD women. Additional covariate analysis was also performed treating absolute GM content as a covariate. Analysis of the relationship between MPFC Glu levels and age, MPFC Glu levels and female hormone concentrations, and BDI scores and group was conducted using the Pearson correlation coefficient. Pearson correlation was used to analyze the relationship between BDI, GCS, and MeRS scores and mean MPFC Glu levels in the PM group. Simple linear regression was used to analyze the impact age had on MPFC Glu levels. Multiple regression analysis controlling for age and %GM was conducted to test whether these factors significantly affected MPFC Glu levels. Cross tabulation analysis of Eta coefficient was used to determine strength of association between participant group and age. One-way analysis of variance (ANOVA) test was used to compare mean MPFC Glu between groups. Statistical significance was defined to be *p* ≤ 0.05.

Metabolite data was analyzed using Cr as a reference molecule to standardize the concentration of the metabolites of interest. As data obtained through PRESS resulted in two separate data point acquisitions per participant, we averaged the adjusted data obtained *via* PRESS for each participant. The exception to this was for the two participants who only had one adequate spectrum for analysis, in which case only one data point was used for analysis. As all data was acquired during one session, results can be validly compared.

## Results

**Figure 3:**
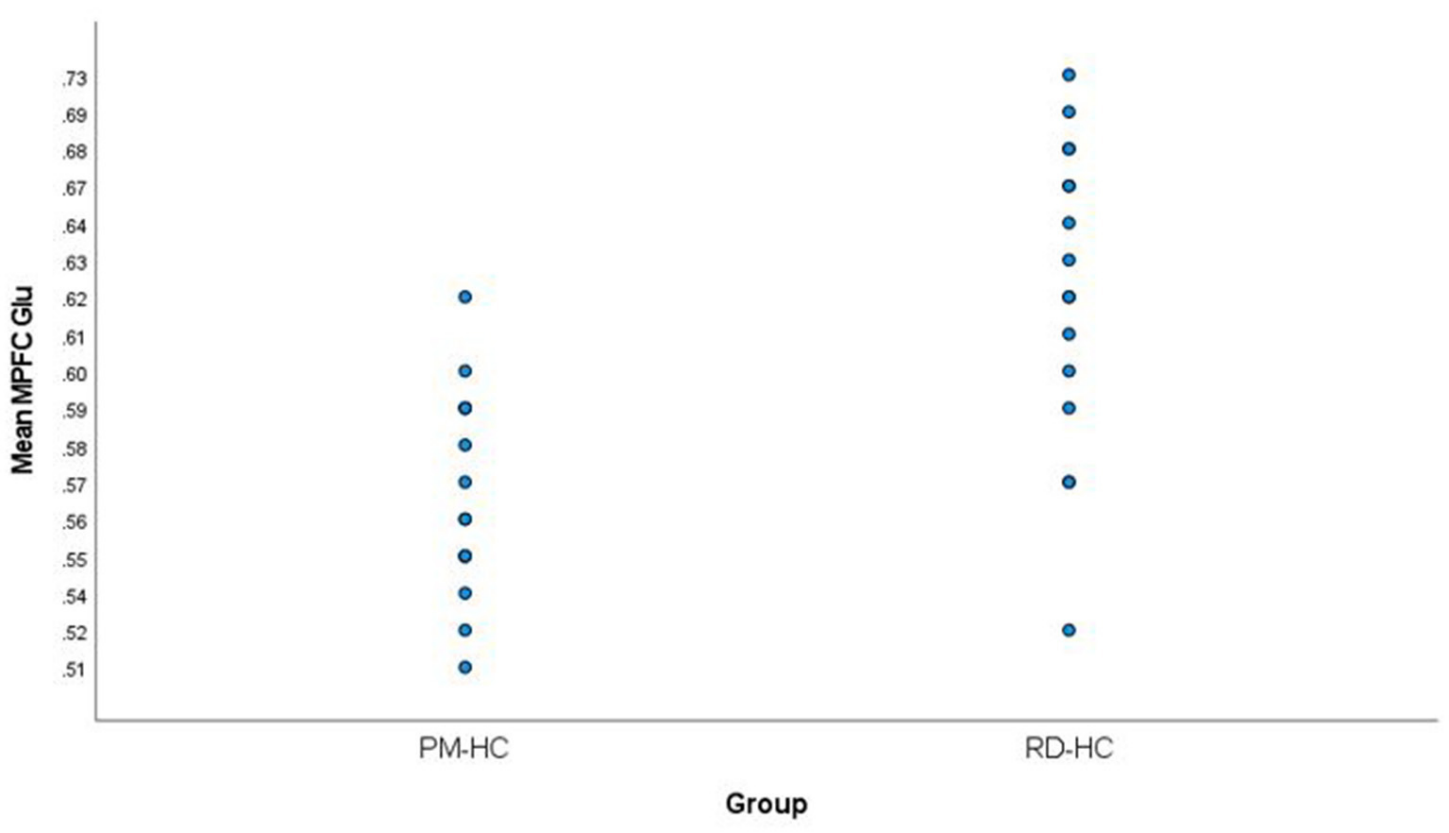
Comparison of mean medial prefrontal cortex creatine-referenced glutamate levels in healthy perimenopausal and reproductive-aged women. MPFC, medial prefrontal cortex; Glu, glutamate; PM-HC, perimenopause healthy controls; RD-HC, reproductive-aged healthy controls.

Medial prefrontal cortex Glu levels were significantly lower in PM participants (0.57 ± 0.03) compared to RD participants (0.63 ± 0.06) (*t* = −3.84, *df* = 23.97, *p* = 0.001) (Figure 3). There were no statistically significant differences in MPFC GM percentage (%GM) and WM percentage (%WM) between PM and RD groups (%GM: 66.33 ± 4.96%, 68.46 ± 5.75, *t* = −1.10, *df* = 29, *p* = 0.28; %WM: 33.67 ± 4.96%, 30.80 ± 5.97, *t* = 1.45, *df* = 29, *p* = 0.16), respectively (Table 1). There was no significant relationship between age and %GM (*r* = −0.34, *df* = 29, *p* = 0.06).

**Table 1:** Creatine-referenced glutamate concentrations and brain tissue composition within the MPFC of healthy PM and RD women.

	**PM participants (*****n*** **=** **15)**		**RD participants (*****n*** **=** **16)**		**Group**	
	**Mean**	**SD**	**Mean**	**SD**	***p*-Value**	***T*** **(d.f. = 29)**
**Metabolite**						
Glu	0.57	0.06	0.63	0.06	0.001^*^	−3.999
%GM	66.33	4.96	68.46	5.75	0.28	−1.10
%WM	33.67	4.96	30.80	5.97	0.16	1.45

*Glu, glutamate; GM, gray matter; MPFC, medial prefrontal cortex; PM, perimenopausal; RD, reproductive; WM, white matter. Brain metabolite measured in institutional units. Metabolite concentration referenced to creatine*.

* *A significant difference between groups*.

As expected, PM women (49.16 ± 3.20 years; range: 44–53 years old) were significantly older than RD women (29.30 ± 9.48 years; range: 18–47 years old) (*t* = 7.82, *df* = 18.58, *p* < 0.001). Correlational analysis found that MPFC Glu levels were significantly related to age with a negative relationship (*r* = −0.64, *df* = 29, *p* < 0.001). Simple linear regression found that aging explained approximately 40% of the variance in MPFC Glu observed [*R*^2^ = 0.40, *F*_(1, 29)_ = 19.69, *p* < 0.001]. This analysis also showed that age significantly predicted a decrease in MPFC Glu levels (β_1_ = −0.003, *p* < 0.001).

Age difference and %GM together significantly explained a portion of the decrease in MPFC Glu observed [*R*^2^ = 0.42, *F*_(1, 29)_ = 10.24, *p* < 0.001], however only age (β = −0.684, *p* < 0.001), and not %GM (β = −0.142, *p* = 0.36), significantly predicted this decrease. Further, when isolating the effects of %GM on MPFC Glu levels, we found that %GM did not significantly explain the decrease in MPFC Glu observed [*R*^2^ = 0.008, *F*_(1, 29)_ = 0.24, *p* = 0.63]. In addition, %GM on its own did not significantly predict a decrease in MPFC Glu levels (β = 0.09, *p* = 0.63).

Due to the nature of overlap between participant group and age, cross tabulation analysis of Eta Coefficient was used to determine strength of association between participant group and age. This analysis revealed that the participant group was strongly associated with age (η = 0.82). Knowing that participant group and age were strongly associated, ANOVA analysis without age correction was used to confirm that mean MPFC Glu levels were significantly lower in PM women compared to RD women [*F*_(1, 29)_ = 15.43, *p* = 0.001]. In summary, there was a decrease in MPFC Glu levels in PM women compared to RD women, however age differences accounted for 40% of the variance observed.

Baseline estradiol (PM: 259.71 ± 306.02; RD: 150.31 ± 57.43; *t* = 1.32, *df* = 13.80, *p* = 0.21) and progesterone (PM: 1.82 ± 0.92; RD: 1.98 ± 1.22; *t* = −0.38, *df* = 28, *p* = 0.70) concentrations were not significantly different between groups. Correlational analysis revealed that MPFC Glu levels were not significantly correlated to either baseline estradiol (MPFC Glu: *r* = −0.19, *df* = 28, *p* = 0.33) or progesterone (MPFC Glu: *r* = −0.09, *df* = 28, *p* = 0.64) concentrations.

Of note, there were cases where hormonal levels were below the detectable range of the assays used. One PM woman (PM group *n* = 14) had estradiol levels below detectable, while four PM (PM group *n* = 10) and five RD women (RD group *n* = 11) had progesterone levels that were below detectable. Excluding these participants from analysis, we found that estradiol (PM: 264.63 ± 302.58; RD: 150.31 ± 57.43; *t* = 1.39, *df* = 13.82, *p* = 0.19) and progesterone (PM: 2.15 ± 0.85; RD: 2.42 ± 1.24; *t* = −0.60, *df* = 20, *p* = 0.56) concentrations were not significantly different between groups. Correlational analysis revealed that MPFC Glu levels were not significantly correlated to either baseline estradiol (MPFC Glu: *r* = −0.22, *df* = 28, *p* = 0.25) or progesterone (MPFC Glu: *r* = −0.04, *df* = 20, *p* = 0.86) concentrations.

Although mean BDI scores were greater in PM (3.60 ± 3.17; range: 10, low score: 0; high score: 10) than RD (1.38 ± 1.78; range: 6; low score: 0; high score: 6) women (*t* = 2.41, *df* = 21.91, *p* = 0.03), scores for both groups were within the range corresponding to depressive symptoms that are considered normal. This indicates that participants were not experiencing any clinically significant depressive symptomology. In addition, mean GCS (5.00 ± 4.69) and MeRS (5.13 ± 4.36) scores indicated that PM participants were experiencing little or no PM-related symptomology (19, 20).

Further correlational analyses were conducted between BDI, GCS, and MeRS scores and mean MPFC Glu levels in the PM group. These analyses revealed no significant correlation between BDI (*r* = 0.12, *df* = 14, *p* = 0.67), GCS (*r* = −0.24, *df* = 14, *p* = 0.38), and MeRS (*r* = −0.26, *df* = 14, *p* = 0.36) scores and mean MPFC Glu levels in the PM group.

## Discussion

To our knowledge, this is the first study measuring MPFC Glu concentrations in PM women. We showed significantly lower MPFC Glu concentrations in PM women.

The issue of the contribution of age to MPFC Glu levels in PM women is a complex one as PM women are inherently older than RD women. Glutamate is mainly found within GM and %GM decreases with age. Had age been the primary factor behind the decrease in MPFC Glu levels in PM women, the decreases in MPFC Glu would have likely been caused by significantly lower %GM in PM women. We did not, however, find any significant differences in %GM between groups, suggesting that the decreased MPFC Glu levels in PM women could not be fully explained by age-induced decreases in %GM in these women who, by definition, are inherently older than RD women. Additionally, linear regression analysis showed that age contributed to 40% of the variance in MPFC Glu concentrations observed, further suggesting that factors other than age contribute to the decreased MPFC Glu levels observed in PM women.

A recent investigation by Lind et al. supports this later statement and the approach we adopted to deal with the age difference between our two groups (21). This study used a 7T magnet to measure Glu levels in the anterior cingulate cortex (ACC), the dorsolateral prefrontal cortex (DLPFC), and the hippocampus in 60 healthy controls separated into three different age groups: a young age group (ages 18–26), a middle age group (ages 39–50), and an older age group (ages 69–79). Our MPFC voxel partially overlapped with the ACC voxel used in Lind et al. As a result, the ACC findings in Lind et al. are relevant to our investigation. Lind et al. found no effect of age on ACC Glu levels and no differences in Glu levels between age groups. This hints at the possibility that the differences in MPFC Glu levels found in our study are unrelated to age and that the association between MPFC Glu levels and age is mostly explained by the fact that PM women are inherently older than women of reproductive age. We must acknowledge that Lind et al. used a different field strength and voxel positioning than our study and included men within their sample. Further research is needed to disentangle the effect of age on MPFC Glu in PM women and to prove that the differences in MPFC Glu levels between PM and RD women are affected by differences in reproductive status.

We suggest that PM status, and related female hormone level fluctuations (22–24), is associated with the decreased MPFC Glu levels observed in PM women. Our research group previously showed that MPFC Glu levels are decreased during the LP compared to the FP of the MC. This phase effect was only found in women who had ovulated, suggesting that the decrease in Glu seen across the MC was the result of hormonal fluctuations associated with ovulation (9). Of note, it has been found that prefrontal cortex Glu levels are lower in females of reproductive age compared to males of similar age (25). It is thus conceivable that differences in female hormonal environment between PM and RD women are contributing to the variations in MPFC Glu levels.

While we suggest that MPFC Glu levels are decreased in PM women due of their reproductive status, we must acknowledge the possibility that an age effect on MPFC Glu levels may be mediated through processes other than an age-induced decrease in %GM. In order to disentangle the effect of age on MPFC Glu in PM women, our group will expand the current study moving forward by including a group of menopausal women in the investigation. These women will be of similar or greater age than the PM women in the current study.

The lack of differences between mean estradiol and progesterone concentrations between groups is not surprising since it has been well-established that concentrations of the hormones estradiol and progesterone should not be used to diagnose PM status (26). During PM, hormone concentrations (especially estrogen) undergo many unpredictable fluctuations in both directions before the total cessation of production by the ovaries. While a general decrease in hormone levels is expected during PM, rapid increases and decreases occur throughout the PM. For example, Santoro et al. found *via* daily urine sampling various daily increases and decreases in female hormone levels in PM women which were not observed in RD women (27). We indeed found a greater standard deviation in estradiol levels in PM women compared to RD women, possibly indicative of rapid fluctuations in estradiol levels exclusive to PM women. Therefore, as female hormone levels rapidly fluctuate during PM, the impact on the glutamatergic system may not be appropriately represented by the concomitant measurements of female hormone concentrations. Furthermore, we did not find a significant correlation between estradiol and progesterone and MPFC Glu levels in the current study. Moreover, female hormones and their metabolites, in addition to their fast neurotransmitter-like activity, also have a delayed impact on transcription which would not be captured by concomitant female hormone measurements. Despite the strong argumentation above, the attribution of decreased MPFC Glu levels to fluctuations in female hormone levels remains to be directly demonstrated.

It is important to note that some PM and RD participants had estradiol and progesterone concentrations that were below the detectable range for the assays used. In these instances, the lowest detectable concentration was used for data analysis (estradiol: 30 pg/ml; progesterone: 1 ng/ml). As this only applied to a small percentage of values, simple substitution was an adequate solution (28). It is possible, however, that this may have led to the overestimation of mean estradiol and progesterone levels.

It is possible that the design of our study impacted our results. We scanned participants during early FP to minimize differences in hormone concentrations, as they are relatively stable at this time compared to other phases of the MC, allowing for more standardized analysis between groups. We may have obtained different results had scanning occurred during a different phase of the MC. Previous research by our lab showed decreases in MPFC Glu during the LP of the MC (9). Not controlling for when scanning occurred and instead having a mixture of data collected from the FP, LP, and anovulatory cycles, however, could have introduced major bias when interpreting data. In addition, planning to scan during the LP would have been impossible for women in late PM as they do not have a regular MC.

A limitation of this study was that sample sizes were relatively small for both PM (*n* = 15) and RD (*n* = 16) groups. Our sample, however, was sufficient to make meaningful comparisons between groups. Further, our previous investigations demonstrating the effect of female hormone fluctuations on MPFC Glu levels were obtained using similar sample sizes to the current study (9, 10), implying a powerful impact of female hormone fluctuations on MPFC Glu levels.

Of the 15 PM participants in total, 11 were identified to be in early PM while the remaining four were in late PM. The increased risk of depression during PM has been particularly linked to late PM (29). While women in early PM are at an increased risk of experiencing MD compared to RD women, this risk is further increased as these individuals' transition to the late PM. Our results may have been different had we only included women identified to be in late PM. Due to the small sample size of PM women, however, we were unable to perform meaningful comparisons between women in early and late PM. Further investigations with greater numbers of women in both early and late PM should be conducted.

Age has been shown to affect Cr in certain brain regions. As we used Cr as the reference molecule for our MPFC Glu measurements, an age effect on Cr would be a potential confounding factor in our study. However, Lind et al. found that the increase in Cr with age in the ACC was only significant when comparing the young age group (ages 18–26) and middle age group (ages 39–50) to the older age group (ages 69–79). When ACC Cr levels were compared between the young and middle age group, no ACC Cr differences were found (21). As the young and middle age group were similar in age to our RD and PM groups, respectively, this indicates that age differences were unlikely to have affected the Cr levels in our study.

A methodological strength of this study was the use of a 3 T magnet for the MRS scans. The use of a magnet with such a field strength allows for higher SNR when compared to magnets of lower strength, allowing for shorter scan times and higher anatomical precision. There are potential limitations to using MRS. First, while PRESS should be able to effectively separate Glu and glutamine signals, it is possible that these signals interfere with each other, preventing a pure Glu signal from being obtained. In general, this is an issue that would typically arise when using a magnet with lower field strength (i.e., 1.5 T magnet). Second, as all obtained metabolite data points were referenced to Cr, there is the possibility of quantification errors. For example, reported differences in metabolite concentrations may instead refer to changes in the concentration of the reference molecule Cr. Despite these concerns, Cr has been used as a reference molecule in previous MRS MD research (30). Further, previous research by our group showed that Cr was not affected by the more acute female hormone changes experienced during pregnancy and post-partum while water, another commonly used reference molecule in MRS research, was affected by the hormonal changes of pregnancy (31). Together, these findings indicate that Cr is a viable reference molecule for MRS research in PM women, who experience comparatively less acute alterations in female hormone concentrations.

In summary, the major finding of our study is that MPFC Glu levels are lower in healthy PM women compared to healthy RD women. Data from other studies suggest that the changes in female hormone levels occurring during PM may be responsible for the decrease in MPFC Glu concentrations. Future studies should compare MPFC Glu levels between PM women suffering from MD and a control group of PM women, with a particular focus on women in late PM.

## Data Availability Statement

The raw data supporting the conclusions of this article will be made available by the authors, without undue reservation.

## Ethics Statement

The studies involving human participants were reviewed and approved by University of Alberta Health Research Ethics Board; University of Alberta. The patients/participants provided their written informed consent to participate in this study.

## Author Contributions

SY contributed to investigation, data analysis, methodology, project administration, writing, and revision of the original draft. JL contributed to investigation, data collection, data analysis, project administration, methodology, reviewing, and editing the manuscript. CH contributed to funding acquisition, data interpretation, reviewing, and editing the manuscript. PS contributed to investigation, data analysis, reviewing, and editing the manuscript. TS contributed to funding acquisition, investigation, reviewing, and editing the manuscript. SH, AL, and HZ contributed to investigation reviewing and editing the manuscript. KA contributed to investigation, data analysis, funding acquisition, reviewing, and editing the manuscript. J-MLM contributed to reviewing, editing the manuscript, funding acquisition, conceptualization, and supervision. All authors contributed to the article and approved the submitted version.

## Funding

This research study was funded through the Cranston Family Grant. AL was supported by the Vessie Heckbert Memorial Summer Research Award. HZ was supported by the ME Ledingham Memorial Summer Research Award. Funding agencies played no role in the design, administration, or interpretation of research data for this study.

## Conflict of Interest

KA declares receipt of two fellowship grants from Janssen Inc., Canada. The remaining authors declare that the research was conducted in the absence of any commercial or financial relationships that could be construed as a potential conflict of interest.

## Publisher's Note

All claims expressed in this article are solely those of the authors and do not necessarily represent those of their affiliated organizations, or those of the publisher, the editors and the reviewers. Any product that may be evaluated in this article, or claim that may be made by its manufacturer, is not guaranteed or endorsed by the publisher.
